# Assessing the Efficacy of Thoracic Erector Spinae Plane Block for Postoperative Analgesia in Lumbosacral Spine Surgery: A Prospective Quasi-experimental Study

**DOI:** 10.7759/cureus.68799

**Published:** 2024-09-06

**Authors:** Jai Prakash Sharma, Uma Devi, Pooja Singh, Sunaina T Karna, Zainab Ahmad, Saurabh Saigal, Ashutosh Kaushal

**Affiliations:** 1 Anesthesiology, All India Institute of Medical Sciences, Bhopal, Bhopal, IND; 2 Neuroanesthesiology and Critical Care, All India Institute of Medical Sciences, New Delhi, New Delhi, IND

**Keywords:** erector spinae plane block, general anaesthesia, general anesthesia+ erector spinae plane block, levobupivacaine, lumbosacral spine surgeries, opioid-sparing, perioperative analgesia, rescue analgesia, ropivacaine, thoracic spine

## Abstract

Background and aims

Optimal postoperative care and analgesia are the key factors in the management of cases of lumbosacral spine surgery. The erector spinae plane (ESP) block is a recently evolving entity and has a dynamic role in postoperative pain management. However, its role in the management of pain in lumber spinal surgeries is still not clear, and the literature remains anecdotal. Therefore, we planned to study the efficacy of ultrasound-guided preoperative ESP block at the T12 level using levobupivacaine for perioperative analgesia in lumbosacral spine surgeries.

Methods

A total of 60 patients scheduled for elective or emergency lumbosacral spine surgery were divided into two groups - the GA group received standard general anesthesia (GA) and the GA+ESP group received standard general anesthesia along with ultra-sound guided ESP block at the T12 level with a bilateral injection of 20 ml 0.25% levobupivacaine. Perioperative analgesia was assessed by total intra-operative fentanyl dose and frequency, intra-operative hemodynamic parameters, post-operative numeric rating scale (NRS) scores, time of first systemic rescue analgesia, tramadol usage, mobilization day, and hospital stay duration.

Results

Intraoperative fentanyl sparing was observed in 83% of the GA+ESP group compared to 33% in the GA group. Postoperative tramadol sparing was observed in 80% of the GA+ESP group compared to 26.7% of the GA group. Twenty-four-hour postoperative NRS scores >3/10 were observed in 20% of the GA+ ESP group compared to 73.3% of the GA group.

Conclusion

In this study, superior perioperative analgesia, opioid-sparing effect, and decreased requirement of postoperative rescue analgesia were observed with ESP block.

## Introduction

Major spine surgery causes severe postoperative pain, which is often difficult to treat. Considering the nature of the surgery, the goal is to tailor the multimodal opioid-sparing analgesic regimen for these patients with stable hemodynamic effects, effective intraoperative and postoperative analgesia, and early recovery with minimal side effects. To deal with severe postoperative pain, systemic analgesics, and epidural and paravertebral blocks have been tried, and their adverse effects and complications impose a concern [1,2].

A novel inter-fascial erector spinae plane (ESP) block was introduced to treat thoracic neuropathic pain [3]. The cadaveric and radiological findings suggested the optimal plane for the ESP block lies deep in the muscle [4]. The erector spinae muscle extends parallel along the thoracolumbar spine, and thus, this permits extensive craniocaudal spread through the thoracolumbar fascia and coverage of multiple dermatomes.

The extensive cranial-caudal spread is a unique advantage of the ESP block, it can be performed at a distance from the surgical field. Furthermore, the observed lack of hindrance in intraoperative electrophysiologic monitoring and the absence of a motor block that might affect postoperative neurologic testing and mobilization are the additional potential advantages of the ESP block [5]. Levobupivacaine is a long-acting s-isomer of the racemate bupivacaine. The duration of action is dose-dependent and varies according to the anesthetic technique while the onset of action of the block is ≤15 mins [6,7]. A meta-analysis concluded that levobupivacaine was more potent than ropivacaine in peripheral nerve blocks [8].

The primary objective of the study was to assess the efficacy of the ESP block on perioperative pain management in terms of its effect on the fentanyl requirement in the intraoperative period and tramadol usage in the first 24 hours of the postoperative period in lumbosacral spine surgery. The secondary objectives were to evaluate the effect of ESP block on intraoperative hemodynamics, postoperative NRS scores, and the time to the first rescue analgesia.

## Materials and methods

This was a single-center, quasi-experimental study conducted by the Department of Anesthesiology over 18 months, after obtaining approval from the institutional human ethics committee of All India Institute of Medical Sciences, Bhopal, with project ID: MD0048 (IHEC-LOP/2019/MD0048). We included 66 patients between 18 and 65 years of age of either sex belonging to the American Society of Anesthesiologists (ASA) grade 1 to 3 undergoing elective/emergency lumbosacral spine surgery. Written and informed consent was obtained from all the subjects.

Participants were allocated into two groups using a quasi-experimental methodology: (1) Group GA: Each patient received standard general anesthesia alone; (2) Group GA + ESP: Each patient received standard general anesthesia plus pre-incisional ultra-sound-guided bilateral erector spinae plane block. 

Exclusion criteria were refusal to block, contraindications to regional technique, e.g., allergy to local anesthetic drugs, infection around the site of block, coagulation disorder, sepsis, pregnancy, and physical or mental health disorders.

All participants received Tab. alprazolam 0.25 mg the night before and on the morning of surgery along with Tab. pantoprazole 40 mg with a sip of water in the morning. In the operation theater, an intravenous (IV) line using an 18-G cannula was established, and standard monitoring, including non-invasive blood pressure, electrocardiogram, pulse oximetry, and capnography, was applied and assessed continuously. 

**Figure 1:**
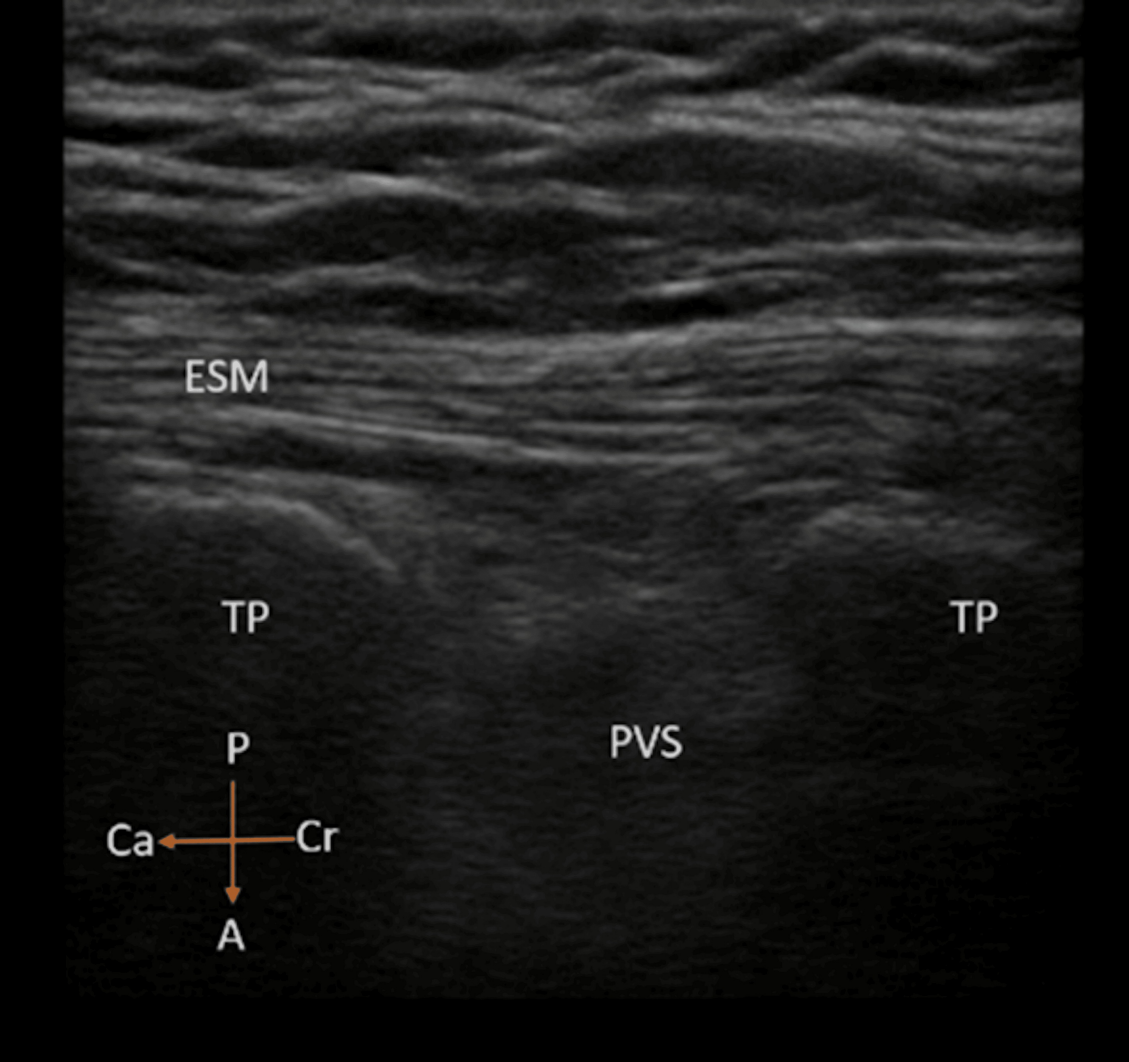
Sonoanatomy of the lower thoracic spine ESM: erector spinae muscle; TP: transverse process, PVS: paravertebral space, Cr: cranial, Ca: caudal, A: anterior, P: posterior

After induction of general anesthesia, participants in the GA+ESP group were given an ESP block in the lateral position under ultrasound guidance. A high-frequency linear transducer (7-13 MHz) of the Sonosite M-Turbo ultrasound machine (Fujifilm, Bothell, WA, US) was placed 3 cm lateral to the vertebral column longitudinally. The transverse processes of the T12 vertebra, the erector spinae, and the psoas muscles were identified (Figure 1).

**Figure 2:**
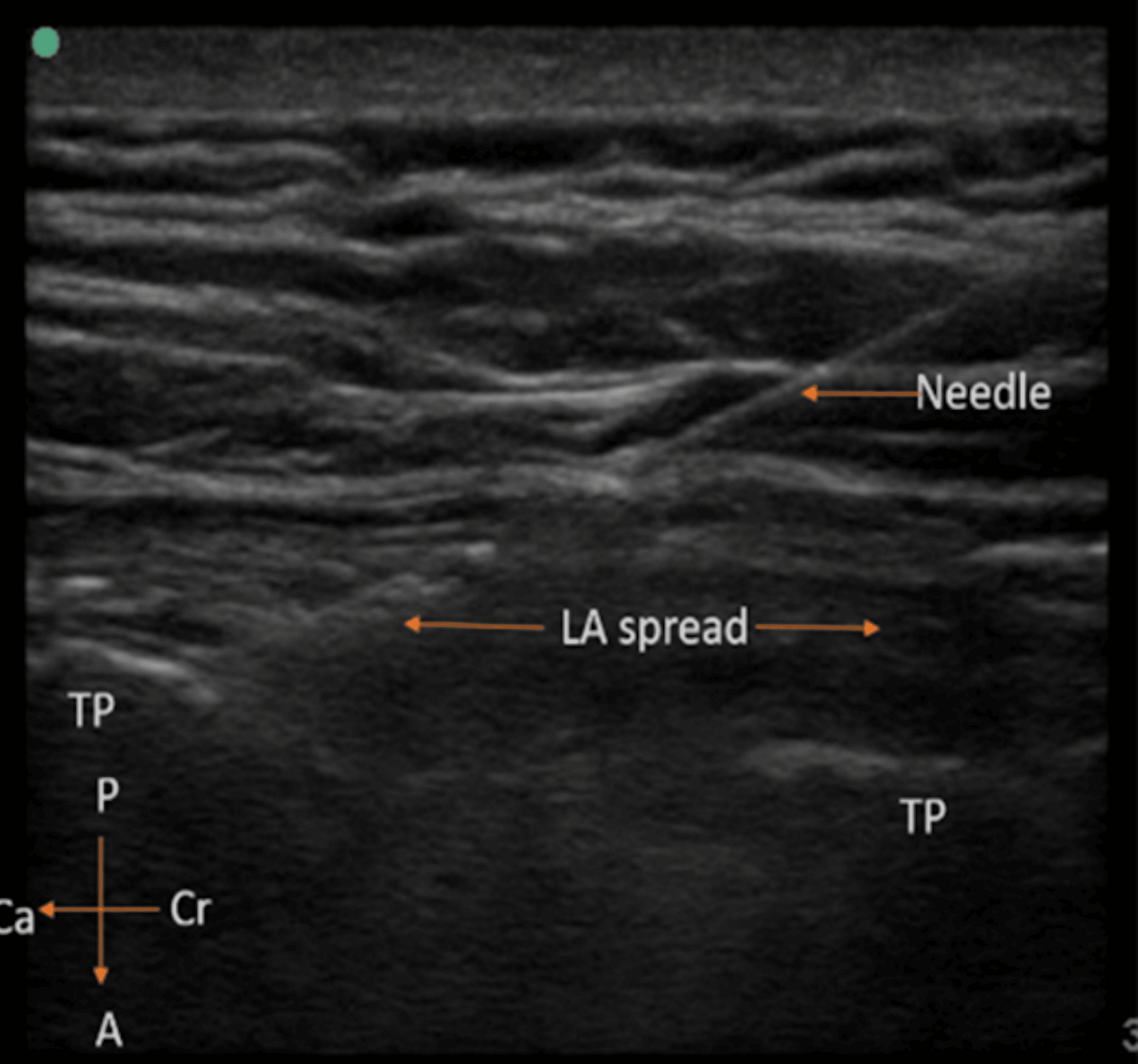
Visualization of the craniocaudal spread of local anesthetic (LA) Cr: cranial, Ca: caudal, A: anterior, P: posterior, TP: transverse process, LA: local anesthetic

Depending on the depth, a 22G ultrasound needle of either 5 or 8cm was inserted through the in-plane technique in a craniocaudal direction until contact was made with the top of the transverse process. After a slight retraction of the needle, normal saline was injected for hydro-dissection to identify the plane, and then 20 ml of 0.25% Inj. levobupivacaine was injected behind the erector spinae muscle (Figure 2).

The same process was repeated on the other side (total dose not exceeding >3 mg/kg). Sensory loss of the posterior dermatomes and dermatomes of the anterior roots of the spinal nerves (lumbar plexus, upper leg) was examined by the blinded examiner every 5 min till 30 min following the block. The patient was observed for any vascular injury, localized swelling, hypotension, and block failure.

All patients were pre-medicated with IV midazolam 0.05 mg/kg and IV fentanyl 1 mic/kg 3 min before the induction of anesthesia. Preoxygenation with FiO2 of 1 was followed by induction with IV propofol 2 mg/kg, once ventilation was ensured, the intubation of the trachea with 7.5 mm ID ET tube was facilitated with IV vecuronium bromide 0.1 mg/kg and followed by a maintenance dose every 30 minutes. Anesthesia was maintained with isoflurane and nitrous oxide with 40% FiO2.

Standard hemodynamic monitoring was done and IV fentanyl 0.5 ug/kg was administered if heart rate, blood pressure, or both increased more than 20% of the baseline. Anesthesia was reversed with IV neostigmine 0.05 mg/kg plus IV glycopyrrolate 0.01 mg/kg, and the trachea was extubated after ensuring an adequate tidal volume and regular spontaneous breathing. Participants were shifted to the post-anesthesia care unit for hemodynamic and numeric rating scale (NRS) score monitoring [9]. Participants were followed up 24 hours postoperatively: NRS scores at 1, 2, 6, 12, and 24 hours, and the time to the first administration of the first dose of tramadol or analgesic was noted. Intravenous (IV) paracetamol 1.0 g infusion was used as the first-line analgesic for NRS scores >3-4/10. If the patient's pain score persisted at >3-4/10 even after one hour of Inj. paracetamol 1 gm, then Inj. tramadol 100 mg was administered as a rescue analgesia IV infusion in 100 ml of normal saline. The time to first administration of analgesia and the total analgesic consumption in the intraoperative period, as well as postoperative, till 24 hours were noted.

Statistical analysis

To detect the mean difference in cumulative analgesia dose of medium effect size with a type 1 error of 5% and power of 80% for the unpaired t-test, we estimated a sample size of 30 in each group. A master sheet was created in Microsoft Excel (Microsoft Corporation, Redmond, WA, US) with the parameters that were measured in the study. Statistical analysis was done on SPSS version 24 (IBM Corp., Armonk, NY, US). Kolmogorov-Smirnov and Shapiro-Wilk normality tests were done to check whether the numerical parameters were distributed normally. Ordinal parameters were compared between the GA and GA+ESP groups using the chi-square and Fisher’s exact tests, and parametric data comparisons were done using an unpaired student’s t-test. Non-parametric numerical parameters were compared using the independent samples' Mann-Whitney U test. A two-way repeated-measures analysis of variance (ANOVA) was performed to see the effect of group and time on NRS scores and intraoperative hemodynamic parameters. Post-hoc pairwise comparisons were done with Bonferroni correction at different time points within the group as well as between the groups. Plotting was done using SPSS version 24 and MATLAB R2018b (MathWorks, Natick, MA, US). p<0.05 was considered significant.

## Results

**Figure 3:**
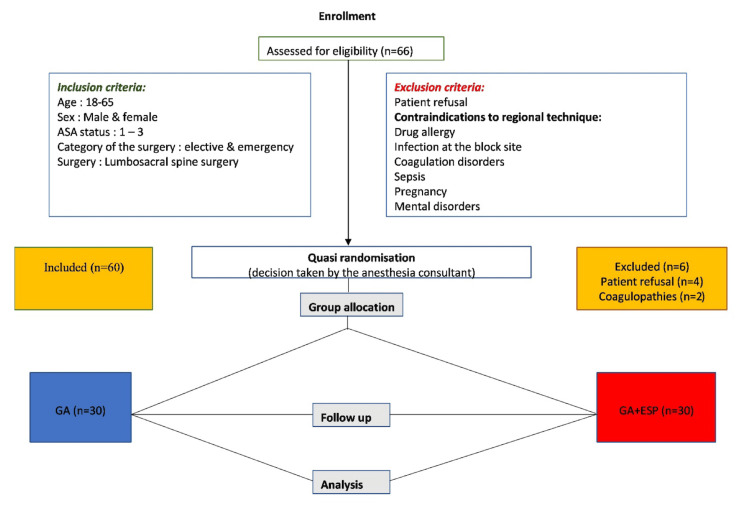
Consolidated Standards of Reporting Trials (CONSORT) flow diagram of the study

The Consolidated Standards of Reporting Trials (CONSORT) flow diagram is shown in Figure 3.

**Figure 4:**
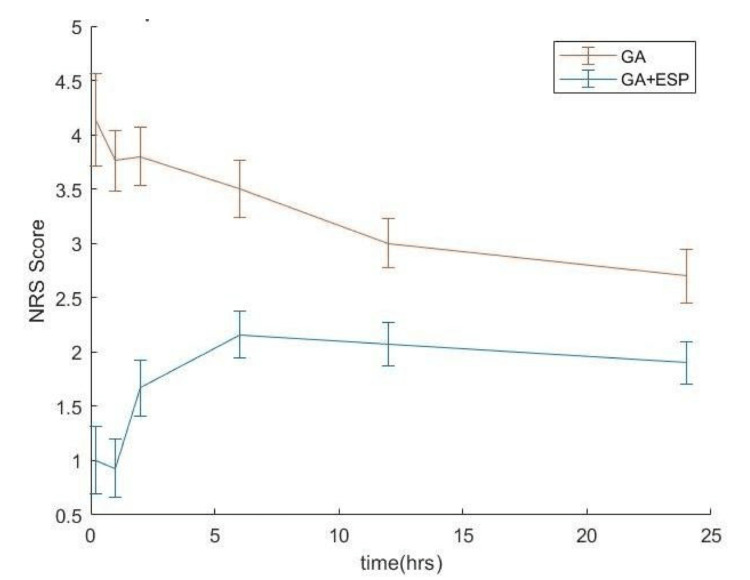
Comparison of NRS scores in the post-operative period at 0,1,2,6,12,24 hr between the two study groups

A comparison of NRS scores in the postoperative period at 0, 1, 2, 6, 12, and 24 hours between the two study groups was done. The median time for the first systemic rescue analgesia was significantly later in the GA+ESP group as compared to the GA group (8 hr vs 2 hr, p-value <0.00925). The NRS scores were significantly higher in the GA group as compared to the GA+ESP group at all time points measured (Figure 4).

Intraoperative heart rate and BP showed a reduction at some time points in the GA+ESP group, but no hemodynamic instability was observed with the use of the block. There were no significant differences in the postoperative day of the first mobilization and duration of hospital stay in the two groups. No block-related complications were noticed.

The two groups were not significantly different in baseline heart rate (HR), systolic blood pressure (SBP), diastolic blood pressure (DBP), and mean arterial pressure (MAP). The primary outcome parameter of intraoperative fentanyl usage was higher in the GA group as compared to the GA+ESP group (66.7% vs 16.7%, p-value <0.000085), and the postoperative tramadol requirement was higher in the GA group than in the GA+ESP group (73.3% vs 20%, p-value <0.00182) (Table 1).

**Table 1:** Comparison of outcome parameters between the two study groups n = number

Outcome parameters	GA (n)	GA+ESP (n)	p-value
Intra-op fentanyl usage: yes/no	20/10	5/25	<0.001
Percentage of patients who required fentanyl during the intraop period	66.7	16.7	
Postop tramadol usage: yes/no	22/8	6/24	
Percentage of patients who required tramadol during the postop period	73.3	20	
The median time for the first systemic rescue analgesia (hr)	2	8	

## Discussion

In this study, most of the GA+ESP group participants receiving a bilateral USG-guided ESP block at the T12 level had low NRS scores compared to the GA group patients at all the time points measured (0, 1, 2, 6, 12, 24 hr). The ESP block provided a good analgesic effect extending to the postoperative period, thereby reducing opioid consumption in the intraoperative as well as the postoperative period, delaying the time for first systemic rescue analgesia [10].

Cadaveric studies revealed that when the local anesthetic is injected deep into the erector spinae muscle, a significant spread of the drug occurs involving the blockade of the dorsal and ventral rami of multiple spinal nerves, thus resulting in sympathetic, visceral, and somatic nerve blocks with a large dermatomal distribution [11]. Providing analgesia by performing the ESP block at a distant location from the injection site has the advantages of minimizing microbial contamination and providing a clear surgical field [12].

Clinical studies have shown that ESP block has been associated with improved analgesic effects in trauma, neuropathies, and postoperative analgesic profiles in breast surgeries, thoracotomies, hip and femur surgeries, ventral hernia repairs, abdominal hysterectomies, thus allowing its usage in a wide range of clinical scenarios at various vertebral levels [13-19].

Lumbar spine surgeries are more invasive with severe postoperative pain, especially on the first postoperative day [20]. Optimal postoperative care and analgesia are key factors in managing spine surgery cases. Though some anaesthesiologists consider epidural analgesia as the gold standard in lumbar spine surgeries for postoperative analgesia, it has its limitations with preoperative placement, surgical interference, and displacement in the postoperative period [21]. The use of opioids has disadvantages like nausea, vomiting, inadequate analgesia, tolerance, drowsiness, sedation, pruritis, urinary retention, constipation, and respiratory depression [22]. Intraoperative use of large bolus doses or continuous infusions of potent opioid analgesics may increase postoperative pain because of their rapid elimination and/or the development of acute tolerance. Hence, it is important to provide non-opioid analgesic agents to treat perioperative pain or opioid-protective anesthesia techniques (techniques to prevent primary and secondary hyperalgesia) to avoid an iatrogenic increase in the intensity of postoperative pain [23].

Few clinical studies have been conducted so far to know the efficacy of erector spinae plane block in spine surgeries. In a similar study with a ropivacaine-based ESP block, there was decreased perioperative opioid usage, enhanced emergence from anesthesia, and early ambulation after surgery [12].

In this study, there was no significant difference in the postop day of first mobilization and duration of hospital stay between the two study groups. This is in contrast to the improved mobilization and reduced hospital stays seen in some previously reported case series [5,12]. Due to the diverse nature of the lumbosacral spine surgeries performed, the operating team, the cause of surgery, and the surgeon’s opinion, no meaningful conclusion about the effect of ESP block on postop mobilization day or length of hospital stay could be made from our study. Reduced opioid consumption was observed in both the groups who underwent micro-lumbar discectomy procedures as compared to more invasive procedures like posterior decompression with pedicle screw fixation and stabilization with rods. This implies that with the increasing invasiveness of the surgery, the chances of muscle ischemia rise, increasing the opioid requirement for better analgesia [24,25].

The sham block is considered for internal validity in terms of blinding, thereby reducing the element of bias. In our study, we have not used a sham block, as injecting an inert substance with no therapeutic value would be considered unethical and expose the patient to unwanted complications [26].

The limitations of our study were: we did not use a sham intervention in the GA group, as operating surgeons were apprehensive about the sham procedure and patients were given a single shot injection of local anesthetic rather than a continuous infusion or intermittent boluses by placing a catheter. The diversity of the surgical procedure can be a confounding factor for the study. The institutional ethics committee also did not allow us to randomize this study.

## Conclusions

In this study, ESP block showed promising results in managing postoperative pain, decreased opioid requirements, and demonstrated stable hemodynamics. A multicentric randomized controlled trial with large sample size is warranted to further establish the efficacy of a thoracic ESP block for postoperative analgesia in lumbosacral spine surgery.
